# Minimally invasive treatment of an amebic empyema secondary to the transdiaphragmatic rupture of a liver abscess: a case report

**DOI:** 10.1093/jscr/rjac334

**Published:** 2022-07-22

**Authors:** Jorge A Abello Vaamonde, Elizabeth Gil White, Alfredo Muñoz López, José M Lorenzo Silva

**Affiliations:** Thoracic Surgery Division, Hospital Español de Mexico, Ciudad de Mexico, Mexico; Thoracic Surgery Division, Hospital Español de Mexico, Ciudad de Mexico, Mexico; Thoracic Surgery Division, Hospital Español de Mexico, Ciudad de Mexico, Mexico; Thoracic Surgery Division, Hospital Español de Mexico, Ciudad de Mexico, Mexico

## Abstract

Liver abscesses are a common complication in patients with amebiasis. Rarely, these will rupture across the diaphragm causing life-threatening empyemas. Evidence justifies performing surgical debridement or decortication for their treatment, given the better overall performance in comparison to open surgeries. However, no current guideline specifies which is the best approach. This report presents the case of a 39-year-old male with clinical, radiographical and microbiological evidence of an amebic empyema secondary to an amebic liver abscess, who received treatment by video-assisted thoracoscopy. The case description highlights the surgical technique, findings and operative outcomes that could be taken into consideration by other physicians to timely manage similar cases. The latter is especially relevant in underdeveloped and developing countries, where the burden of amebiasis appears to be greater. To the best of the authors’ knowledge, this is the first description of a transdiaphragmatic amebic infection treated in a minimally invasive fashion.

## INTRODUCTION

Amebiasis, a protozoan infection prevalent in 50% of subtropical populations, complicates to amebic liver abscess (ALA) in 2–5% of cases. If left untreated, transdiaphragmatic rupture followed by direct dissemination of amebiasis may result in a life-threatening empyema. Although this complication is rare, occurring only in 2–3% of invasive cases, mortality ranges from 5 to 100% depending on the effectiveness of the treatment provided [1, 2]. Currently, there is no guideline specifying the ideal intervention for these cases. According to the Surgical Case Reports (SCARE) Statement [3], we present a case report of pleuropulmonary amebiasis due to ruptured ALA treated in a minimally invasive fashion.

## CASE REPORT

A 39-year-old male with no relevant history attends an emergency department (ED) in Mexico City after a 22-day history of asthenia, low-grade fever, arthralgias, myalgias and odynophagia. SARS-CoV-2 PCR tests were negative at Days 7 and 12. Despite empiric antibiotic therapy with azithromycin, the patient suffered worsening of symptoms and increasing right upper quadrant pain irradiated along the right arm. A primary care doctor recommended modifying current antimicrobial treatment for amoxicillin-clavulanate, which after completion was also ineffective. On Day 19 the patient noted his eyes were turning yellow, prompting additional external evaluation and subsequent ED referral.

**Figure 1:**
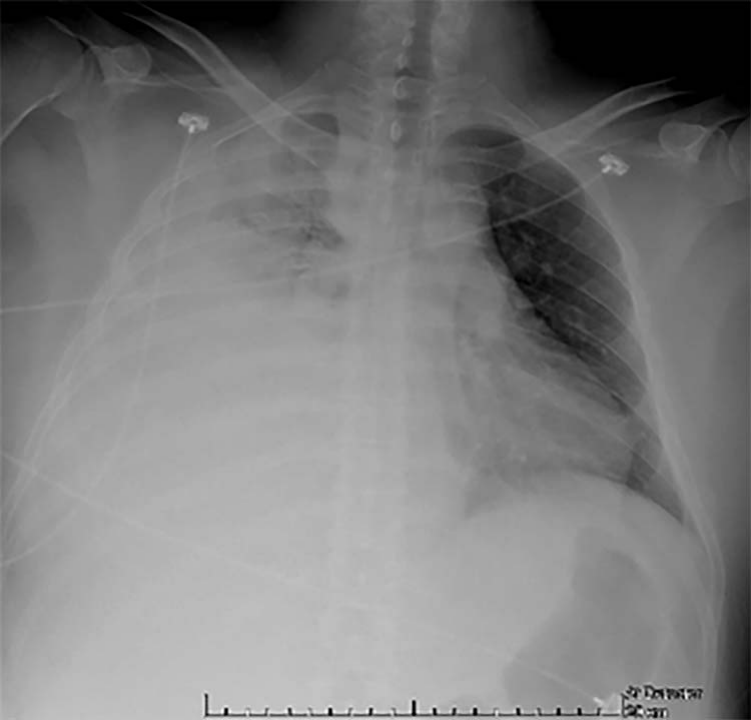
CXR at admission: right pleural effusion occupying 90% of the lung.

**Figure 2:**
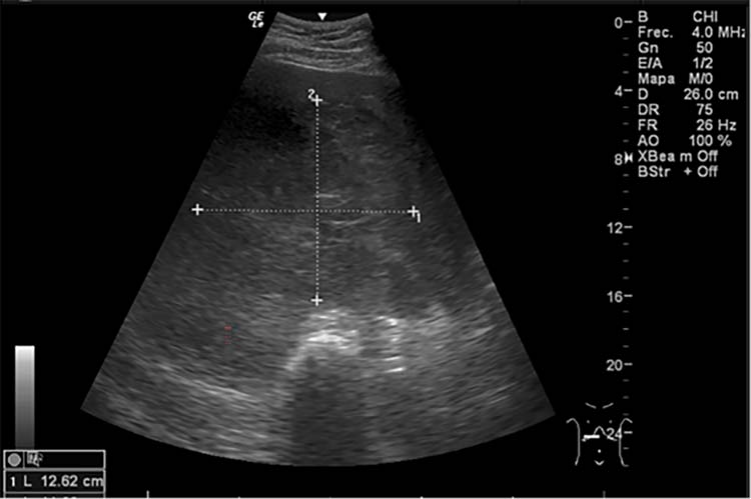
Abdominal US at admission: nodular lesion in the right liver lobe.

**Figure 3:**
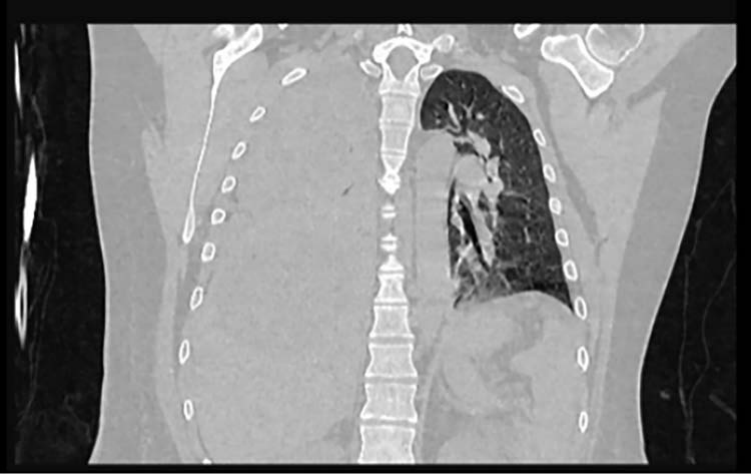
Frontal chest CT at admission: right pleural effusion occupying 100% of the lung.

**Figure 4:**
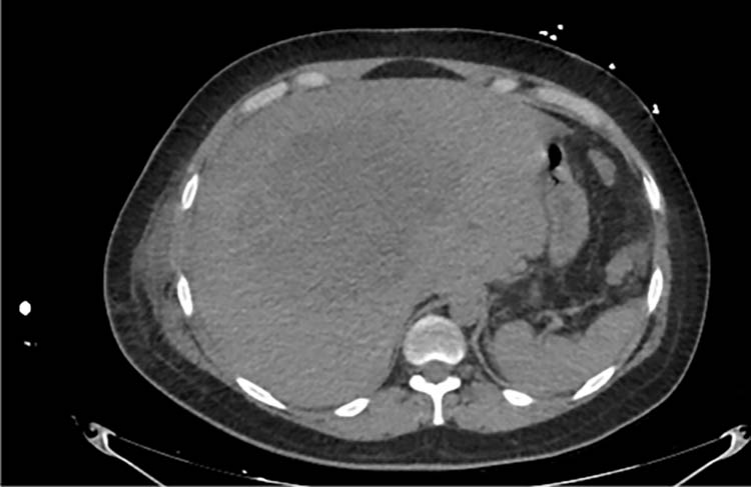
Axial abdominal CT at admission: 20 cm nodular lesion occupying the right liver lobe.

Examination revealed tachycardia, tachypnea, jaundice, dehydration, jugular vein distension, right lung hypoventilation, ascites, upper abdominal tenderness and bilateral lower limb edema. Laboratory workup highlighted neutrophilic leukocytosis, with WBCs exceeding 20 000/μl, along with an abnormal hepatic function panel. The latter showed hypertransaminasemia with ALT predominance over AST (344.7 U/L and 288.5 U/L, respectively), hyperbilirubinemia (10.01 μmol/L) and elevated values of ALP (909.1 U/L) and DHL (709.9 U/L). Of relevance, creatinine elevation (2.11 mg/dl), thrombocytosis (629 × 10^9^/L) and positive D-Dimer (5780 ng/ml) were also noted, so acute kidney injury management and thromboprophylactic measures were initiated. A chest X-ray (CXR) showed a massive right pleural effusion (Fig. 1) and an abdominal ultrasound (US) revealed hepatomegaly with a right nodule (Fig. 2). After further evaluation, tomographic evidence of a right pleural effusion occupying 100% of the lung (Fig. 3) and a 20 cm hypodense, nodular lesion in the right liver (Fig. 4) were identified. These findings were suggestive of an ALA concomitant with an empyema. The patient was admitted into the intensive care unit and prepared for surgery.

While performing a uniportal video-assisted thoracoscopic surgery (uVATS), 1 L of free anchovy-paste-like exudate was found within the cavity, which after analysis was reported positive for *Entamoeba histolytica*. Also, loss of diaphragmatic tissue continuity and pulmonary fibropurulent adherences were noted, prompting pleural decortication and diaphragmatic debridement. An unexpected pericardial bleeding made conversion to lateral thoracotomy necessary in order to achieve hemostasis with monopolar energy. After, once drainage of abnormal fluid was completed, a Jackson Pratt drain was placed across the ruptured diaphragm and the thoracic cavity was closed. The drainage initially consisted of a brown-red fluid with abundant sediment but normalized in the following days. Although postoperative CXR showed a right pleural effusion of 30%, no complications occurred immediately after surgery. The patient was readmitted to the ICU and 750 mg of metronidazole in combination with 2 g of cefepime were administered every 8 and 12 h, respectively.

**Figure 5:**
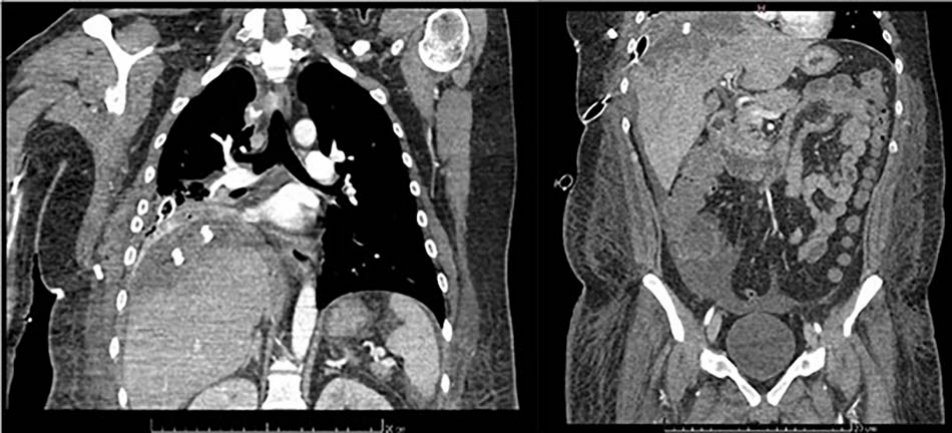
Frontal thoraco-abdominopelvic CT at 9 days after surgery: subpleural collection of 79 × 34 mm, heterogenous, hypodense hepatic lesion with contrast enhancement on its borders, fluid collection around the liver, paracolic gutters and pelvic cavity.

Follow-up was uneventful until the ninth day. Although laboratory parameters were normalizing, a contrasted computed tomography (CT) scan showed a subpleural collection of 79 × 34 mm, as well as an heterogenous, hypodense hepatic lesion with wall enhancement (Fig. 5). So, an additional VATS was performed through the anterior incision of the previous pleural tube. Adhesions were removed and a 24 FR Blake drain was positioned at the postero-apical region of the cavity. Other than small amounts of serosanguineous drainage, no relevant findings or events were documented until 9 days after such reintervention. By then, an abdominal US revealed a newly formed abscess on the right convexity of the liver measuring 86.5 cc and containing both hyperechoic and hypoechoic contents, and a 2.6 cm nodule on the V hepatic segment. As there were no clinical or biochemical repercussions, conservative management was initiated and completed successfully.

Given clinical stability, chest tubes were removed, and discharge was signed on Day 32 after admission.

## DISCUSSION

After fecal-oral transmission of amebic cysts, trophozoites invade the colonic wall and reach the portal circulation, facilitating hepatocyte necrosis and ALA formation [4]. Within less than a month, patients develop signs and symptoms suggestive of such disease. Of note, jaundice characterizes <10% of cases, as opposed to its high prevalence in patients with pyogenic abscesses. Empiric treatment with Metronidazole, Tinidazole or Paromomycin is reasonable if the patient meets epidemiological, clinical and radiographic criteria [5]. However, in refractory cases or under high risk of rupture, therapeutic needle aspiration or percutaneous drainage should be considered [6].

If untreated, transdiaphragmatic rupture may occur causing pleuropulmonary amebiasis. According to the European Association for Cardio-Thoracic Surgery, fibrinopurulent empyemas limit lung re-expansion, thus requiring surgical debridement or decortication over tube thoracostomy [7]. A video-assisted approach has similar disease-resolution rates when compared with open surgery, but also achieves less blood loss, decreased postoperative pain, shorter length of hospital stay and fewer complications [8]. Complete VATS (cVATS), which consists of three to four ports, has evolved to uVATS, which consists of a single port. Van Middendorp et al. [9] compared both techniques and found no significant difference regarding surgical outcomes. However, procedural duration of uVATS was longer compared with that of cVATS. Based on experience, the authors expect that after the learning curve, this tendency could be reversed. Regardless of the video-assisted approach, conversion to open thoracotomy is performed if resolution of the empyema and lung expansion is not adequately achieved. Previous studies have concluded that increased time between symptom onset and VATS is directly associated with conversion to open thoracotomy, especially after the second week, making early referral and management essential for these patients [10].

## CONCLUSION

Given the lethal consequences of untreated ALA transdiaphragmatic rupture, thoracic surgeons must take into consideration the value of timely video-assisted rather than open procedures to treat their patients. This is of significant relevance in underdeveloped and developing countries, where the burden of amebiasis appears to be greater.
